# The Synergistic Effect of Reduced Graphene Oxide and Proteasome Inhibitor in the Induction of Apoptosis through Oxidative Stress in Breast Cancer Cell Lines

**DOI:** 10.3390/ijms25105436

**Published:** 2024-05-16

**Authors:** Rafał Krętowski, Beata Szynaka, Agata Jabłońska-Trypuć, Anna Kiełtyka-Dadasiewicz, Marzanna Cechowska-Pasko

**Affiliations:** 1 Department of Pharmaceutical Biochemistry, Medical University of Bialystok, Mickiewicza 2A, 15-222 Białystok, Poland; marzanna.cechowska-pasko@umb.edu.pl; 2 Department of Histology and Embryology, Medical University of Białystok, Waszyngtona 13, 15-269 Białystok, Poland; beataszynaka@gmail.com; 3 Department of Chemistry, Biology and Biotechnology, Faculty of Civil Engineering and Environmental Sciences, Bialystok University of Technology, Wiejska 45E Street, 15-351 Białystok, Poland; a.jablonska@pb.edu.pl; 4 Department of Plant Production Technology and Commodity, University of Life Sciences in Lublin, 20-950 Lublin, Poland; anna.kieltyka-dadasiewicz@up.lublin.pl; 5 Garden of Cosmetic Plants and Raw Materials, Research and Science Innovation Center, 20-819 Lublin, Poland

**Keywords:** apoptosis, breast cancer, cytotoxicity, oxidative stress, reduced graphene oxide (rGO), proteasome inhibitor (MG-132)

## Abstract

Reduced graphene oxide (rGO) and a proteasome inhibitor (MG-132) are some of the most commonly used compounds in various biomedical applications. However, the mechanisms of rGO- and MG-132-induced cytotoxicity remain unclear. The aim of this study was to investigate the anticancer effect of rGO and MG-132 against ZR-75-1 and MDA-MB-231 breast cancer cell lines. The results demonstrated that rGO, MG-132 or a mix (rGO + MG-132) induced time- and dose-dependent cytotoxicity in ZR-75-1 and MDA-MB-231 cells. Apart from that, we found that treatment with rGO and MG-132 or the mix increased apoptosis, necrosis and induction of caspase-8 and caspase-9 activity in both breast cancer cell lines. Apoptosis and caspase activation were accompanied by changes in the ultrastructure of mitochondria in ZR-75-1 and MDA-MB-231 cells incubated with rGO. Additionally, in the analyzed cells, we observed the induction of oxidative stress, accompanied by increased apoptosis and cell necrosis. In conclusion, oxidative stress induces apoptosis in the tested cells. At the same time, both mitochondrial and receptor apoptosis pathways are activated. These studies provided new information on the molecular mechanisms of apoptosis in the ZR-75-1 and MDA-MB-231 breast cancer cell lines.

## 1. Introduction

Breast cancer (BC) is the most commonly diagnosed cancer in women worldwide. More than 2 million new cases of BC were reported in 2020. Moreover, the morbidity and mortality due to this cancer have increased over the last few years. Epidemiological studies have shown that approximately 80% of BC patients are >50 years old. Breast cancer can be classified into several molecular subtypes (Luminal A, Luminal B, HER2-enriched and basal-like). BC treatment is complex and multi-stage. It includes surgery, radiotherapy, chemotherapy, hormone therapy or biological therapy performed in various sequences [1].

The balance between cell proliferation and apoptosis is important for maintaining homeostasis in the human body. Disruption of this balance leads to numerous diseases, including autoimmune and neurodegenerative diseases and cancer [2].

The ubiquitin–proteasome pathway is responsible for the degradation of proteins with a short half-life. Additionally, it plays an important role in regulating many different cellular processes, including cell cycle progression, differentiation and apoptosis. Many proteins present in the cell are tightly regulated by the proteasome, such as tumor inhibitors (P53, E2A, c-Myc, c-Jun, c-Fos), transcription factors (NF-kB, IkBa, HIFI, YYI, ICER) and proteins actively involved in cell cycle regulation (cyclin A and B, P27, P21, IAP1/3) or apoptosis [2].

The ubiquitin–proteasome pathway is closely related to apoptosis. The proteasome, a multi-catalytic enzyme complex present in the nucleus and cytoplasm of cells, is involved in the degradation of misfolded proteins previously labeled with ubiquitin. Ubiquitinated proteins are recognized by the 26S proteasome, which hydrolyzes and degrades ubiquitin-tagged proteins into low-molecular-weight degradation products. The 26S proteasome consists of a 20S core (700 kDa), in which the labeled proteins are digested into short peptides, and two 19S regulatory complexes (900 kDa) each. They are made up of many (15–20) different subunits. Six of them have ATPase activity. These subunits participate in the unfolding of proteins at the expense of energy released from ATP. There are three types of proteolytic activity within the 20S core particle: caspase-like, trypsin-like and chymotrypsin-like activity. Blocking proteasome activity leads to a number of toxic effects, including an increase in reactive oxygen species (ROS) production; glutathione (GSH) depletion due to proteasome inhibitors can cause mitochondrial dysfunction and caspase-8 or caspase-9 activation, leading to loss of cell viability [3]. Oxidative stress is defined as a complex and dynamic process caused by excessive production of ROS that the cell cannot effectively remove. Oxidative stress can lead to damage to macromolecules such as lipids, proteins or nucleic acids. Additionally, this process activates signal transduction pathways that induce programmed cell death—apoptosis. Proteasomes actively participate in the regulation of the activities of antioxidants, catalase, heme oxidase-1 (HO-1) and superoxide dismutase [4].

Blocking proteasome activity leads to the accumulation of unfolded and/or damaged proteins in the cells. As a result of the inhibition of proteasome activity, the cell activates mechanisms adapting it to unfavorable microenvironmental conditions, which include unfolded protein response (UPR), autophagy and, in the case of severe damage, apoptosis. Peptide aldehyde proteasome inhibitor MG-132 (carbobenzoxyl-L-leucyl-L-leucyl-L-leucinal) is a natural triterpene proteasome inhibitor derived from a Chinese medicinal plant and is able to suppress the growth of human prostate cancer in nude mice. MG-132 inhibits the activity of the 20S proteasome by covalently binding to the active site of the beta subunit, thereby effectively blocking its proteolytic activity. MG-132 blocks the proliferation of cancer cells as a result of cell cycle arrest or induces the activation of the apoptosis process [5].

On the other hand, graphene oxide (GO) and its reduced form (rGO), multi-layer graphene, ultrathin graphite and graphene nanosheets also belong to the family of graphene nanomaterials (GFNs) with proven pro-apoptotic effects. Differences in the molecular structure or physical properties of GO and rGO depend largely on the synthesis method. Graphene oxide is a highly oxidized chemical form of graphene and rGO. Moreover, rGO is known to be more hydrophobic and cytotoxic than GO. Reduced graphene oxide nanoparticles have been widely used in biomedical applications, such as biosensing/bioimaging, disease diagnosis, gene and drug delivery, cancer therapy, photothermal therapy bacterial inhibition, antibacterial papers, antiviral materials and tissue engineering [6,7]. Apart from that, depending on the size, oxidation state and concentration of nanoparticles and the experimental model used, graphene, GO and rGO show different levels of cytotoxicity [8].

In our previous work, we demonstrated the effectiveness of rGO as a pro-apoptotic agent [9]. In turn, literature data indicate the anticancer effect of a proteasome inhibitor (MG-132) [10]. An innovative, previously undemonstrated aspect of this work is the combination of these two effective factors, which can not only enhance the pro-apoptotic effect, but also possibly reduce the level of toxicity of these compounds in the body, while simultaneously demonstrating anticancer activity. 

Despite research on the cytotoxicity of rGO and MG-132, there are no studies on the effects of both compounds administered simultaneously. This is a key aspect of the novelty of this study and will allow for in vivo research and the development of innovative methods of fighting breast cancer.

The purpose of this study was to investigate the influence of rGO and MG-132 on viability, apoptosis and oxidative stress in human breast cancer ZR-75-1 and MDA-MB-231 cell lines. We hypothesized that rGO and MG-132 induce strong cytotoxic effects via oxidative stress on both breast cancer cell lines. Using these results, we sought to determine whether it is possible to identify the characteristics of rGO and MG-132 that might be useful in anti-breast-cancer therapy. Moreover, our studies indicate two possible paths for the induction of apoptosis induction in breast cancer cells by rGO and MG-132.

## 2. Results

### 2.1. The Effect of rGO, MG-132 or rGO with MG-132 on the Viability of Breast Cancer Cells

**Figure 1:**
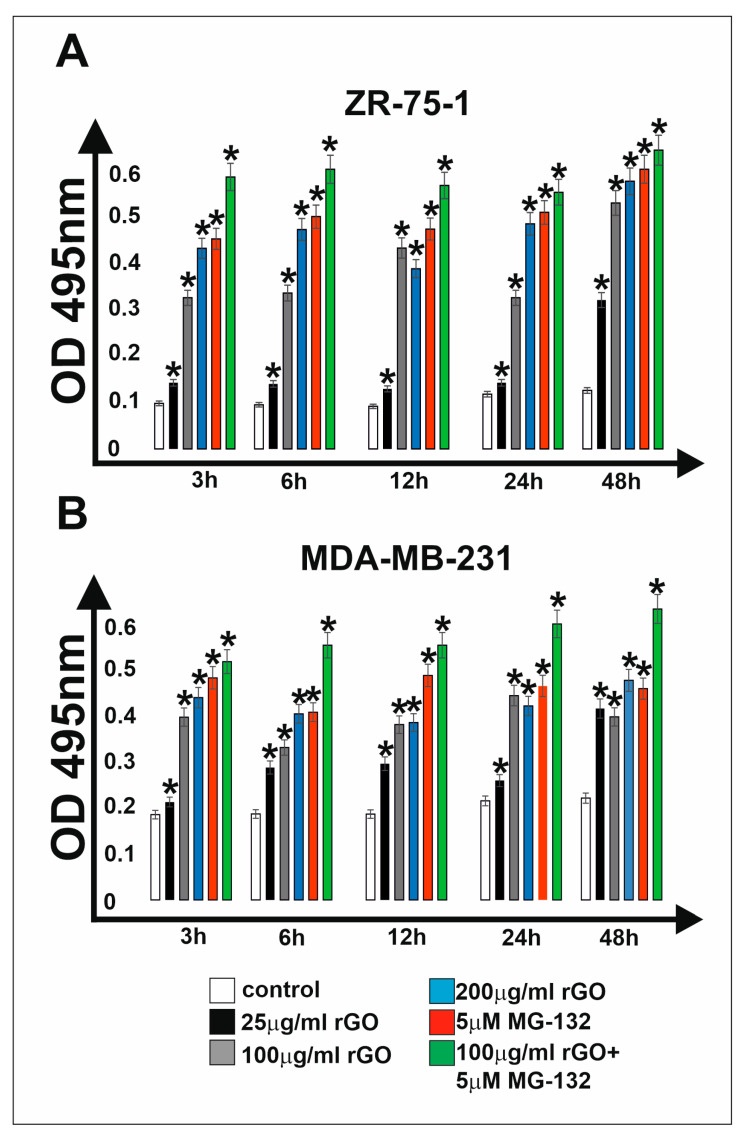
The effect of rGO, MG-132 and rGO with MG-132 on the release of LDH into the culture medium of ZR-75-1 (**A**) and MDA-MB-231 (**B**) cells. Cells were incubated with increasing concentrations of rGO (25 µg/mL, 100 µg/mL, 200 µg/mL) or one concentration of MG-132 (5 µM) or a mix consisting of rGO (100 µg/mL) with MG-132 (5 µM) for 3 h, 6 h, 12 h, 24 h and 48 h. Mean values from three independent experiments ± SD are presented. * *p* < 0.05 represents significant effects between treatments and control followed by a Dunnett’s test.

The cytotoxic effects of rGO, MG-132 or rGO with MG-132 on breast cancer cell lines ZR-75-1 (Figure 1A) and MDA-MB-231 (Figure 1B) were examined by LDH assay (Figure 1A,B). During our experiment, both breast cancer cell lines were incubated with increasing concentrations of rGO (25–200 µg/mL), one concentration of MG-132 (5 µM) or a mix consisting of rGO (100 µg/mL) with MG-132 (5 µM) for different time intervals (from 3 h to 48 h). We observed that rGO caused a time- and dose-dependent increase in LDH release into the culture medium of both breast cancer cell lines. This indicates increased cytotoxicity of rGO on the tested breast cancer cells. Increased LDH release in both breast cancer cell lines was observed after 3 h, 6 h, 12 h, 24 h and 48 h of incubation with all concentrations of rGO used. Prolongation of the incubation time to 48 h in both breast cancer cells resulted in a strong release of LDH to the culture medium. One concentration of rGO (100 µg/mL) was selected for further analyses. Moreover, during the incubation of cells with MG-132 (5 µM), a time-dependent increase in LDH release by ZR-75-1 and MDA-MB-231 cells into the culture medium was observed. Interestingly, in breast cancer cells treated with a mix consisting of rGO (100 µg/mL) with MG-132 (5 µM), the LDH-releasing effect was more pronounced compared to cells incubated with rGO (100 µg/mL) or MG-132 (5 µM) separately.

### 2.2. The Effect of rGO, MG-132 and rGO with MG-132 on Biochemical Parameters of Oxidative Stress

**Figure 2:**
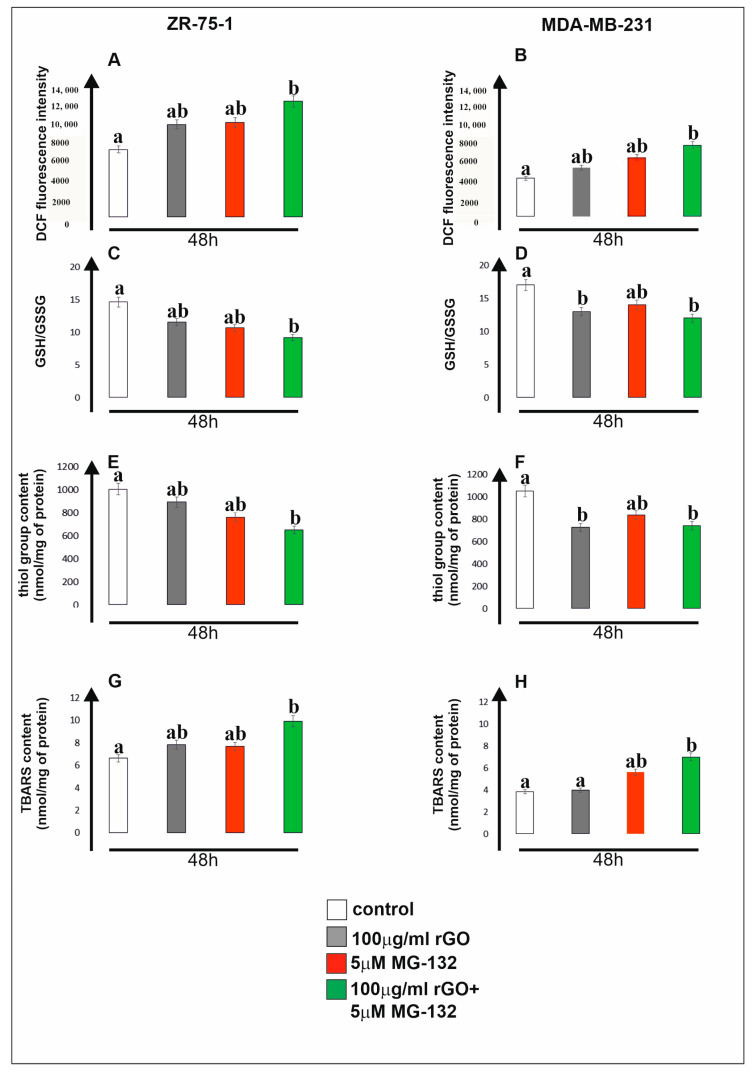
Biochemical parameters of oxidative stress induced via rGO, MG-132 or rGO with MG-132 in ZR-75-1 and MDA-MB-231 cells. Cells were incubated with rGO (100 µg/mL), MG-132 (5 µM) or a mix consisting of rGO (100 µg/mL) with MG-132 (5 µM) for 48 h. Intracellular synthesis of reactive oxygen species (ROS) in ZR-75-1 and MDA-MB-231 cells is presented in panels (**A**,**B**). Panels (**C**,**D**) show the GSH/GSSG ratio. The intracellular thiol group content is shown in panels (**E**,**F**), while panels (**G**,**H**) show the TBARS (thiobarbituric acid reactive substance) content. Mean values from three independent experiments ± SD are presented. Different letters indicate statistical differences (*p* ≤ 0.05) between treatments estimated by Tukey’s test.

In the present study, we observed high levels of ROS, measured by DCF fluorescence intensity, in both analyzed cell lines (Figure 2, panels A and B). A significantly higher level of ROS characterized the ZR-75-1 cell line (panel A). However, it should be emphasized that in both cell lines, we noted an increase in the tested parameter compared to untreated control cells. A particularly statistically significant increase in ROS content was noted under the influence of rGO (100 µg/mL) with MG-132 (5 µM), both in relation to the control and in relation to the cells exposed to rGO and MG-132 alone. This indicates a strong induction of ROS synthesis in cells treated simultaneously with the two tested compounds. 

A similar trend showing an increase in the level of oxidative stress can be noticed when analyzing changes in the level of the GSH/GSSG ratio (Figure 2, panels C and D). In both cell lines exposed to rGO and MG-132, a significant reduction in the intracellular concentration of GSH, which is one of the key low-molecular-weight antioxidants, was observed. In the ZR-75-1 cell line (panel C), the combination of both tested compounds was most effective, similar to what was observed for the MDA-MB-231 cell line (panel D). Lowering glutathione levels is indicated in cancer cells because it increases their sensitivity to chemotherapy. 

Glutathione, together with the -SH groups of proteins, constitutes the most important thiol buffer of cells, and the ratio of its reduced form to its oxidized form is a measure of the oxidation–reduction status of cells. In our studies, we showed a statistically significant decrease in the level of -SH groups compared to the control in both tested cell lines (Figure 2, panels E and F). Particularly significant is the decrease observed as a result of exposure of cancer cells to the combination of rGO and MG-132. It should be emphasized that it is recorded both for the control and for both tested compounds used alone. 

Typically, a decrease in the level of -SH groups is accompanied by an increase in the level of membrane lipid peroxidation. The end products of the lipid peroxidation process are carcinogenic and may affect cell proliferation. The obtained results show that the mix of rGO and MG-132 clearly stimulates this process compared to the control (Figure 2, panels G and H). Also in this case, none of the tested compounds alone was as effective as their combination. Similar results were obtained for both tested cell lines, but it is worth noting that the TBARS (thiobarbituric acid reactive substance) content was significantly higher in the ZR-75-1 line than in MDA-MB-231 (Figure 2, panels G and H).

### 2.3. The Effect of rGO, MG-132 and Mix of rGO with MG-132 on Antioxidant Enzymes Such as GPx and SOD

**Figure 3:**
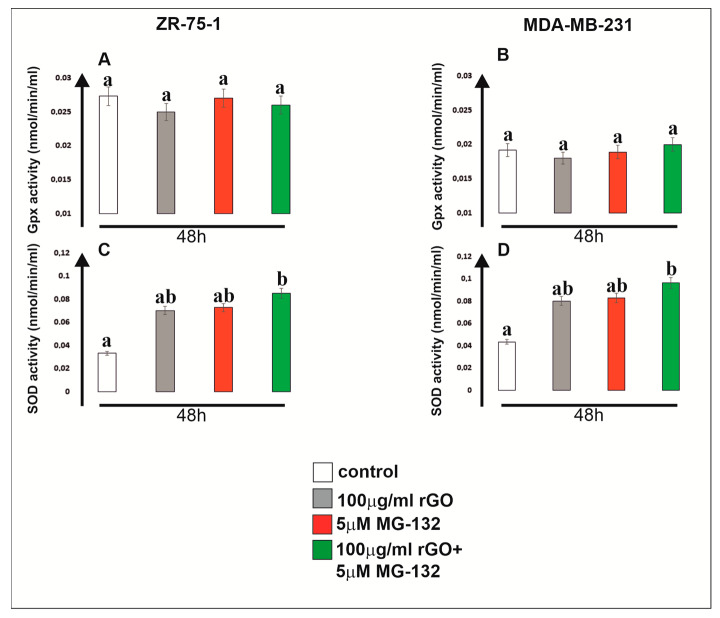
The effect of rGO, MG-132 and the mix (rGO with MG-132) on GPx (glutathione peroxidase) activity (**A**,**B**) and SOD (superoxide dismutase) activity (**C**,**D**) in ZR-75-1 and MDA-MB-231 cells. The cells were cultured with compounds for 48 h. Mean values from three independent experiments ± SD are shown. Different letters indicate statistical differences (*p* ≤ 0.05) between each treatment estimated by Tukey’s test.

We observed a higher activity of glutathione peroxidase (GPx) in ZR-75-1 cells than in the MDA-MB-231 cell line. Apart from that, we did not find a statistically significant increase in GPx activity compared to the control cells in both breast cancer cell lines (Figure 3, panels A and B). However, it should be emphasized that in both cell lines, we noted an increase in activity of superoxide dismutase (SOD) compared to untreated control cells. A particularly statistically significant increase in SOD activity was noted under the influence of rGO (100 µg/mL) with MG-132 (5 µM), both in relation to the control and to the cells exposed to rGO and MG-132 alone (Figure 3, panels C and D). This indicates a strong induction of oxidative stress in cells treated simultaneously with the two tested compounds.

### 2.4. The Effect of rGO, MG-132 and rGO with MG-132 on Apoptosis and Necrosis

**Figure 4:**
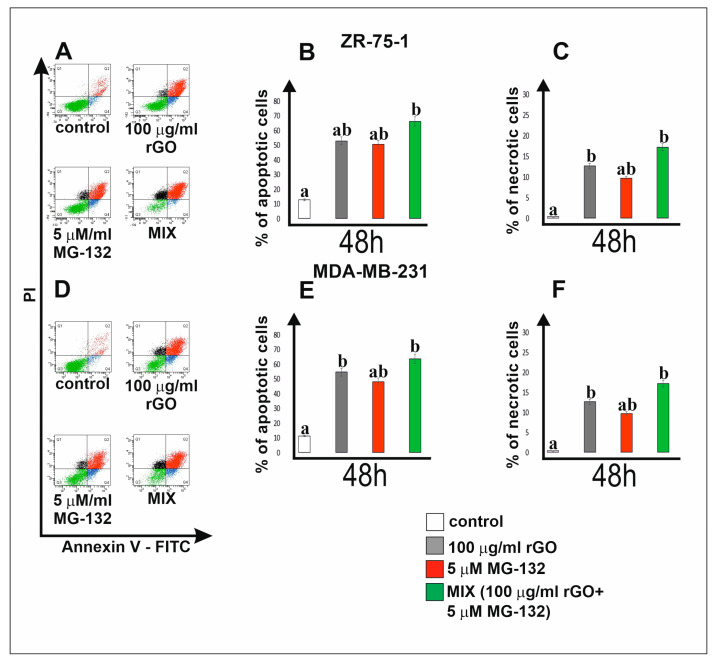
Effect of rGO, MG-132 or a mix consisting of rGO and MG-132 on apoptosis and necrosis of ZR-75-1 (panels **A**–**C**) and MDA-MB-231 (panels **D**–**F**) cells. Both breast cancer cell lines were incubated for 48 h. The percentage of apoptotic (panels **A**,**B**) and necrotic (panels **D**,**F**) cells was assessed by flow cytometry with Annexin V-FITC and propidium iodide (PI) double-staining. The sum of quadrants Q2 and Q4 represents the percentage of apoptotic cells, while quadrant Q1 represents the percentage of necrotic cells in a representative flow cytometry bar graph analysis. Mean values from three independent experiments ± SD are presented. Different letters indicate statistical differences (*p* ≤ 0.05) between treatments estimated by Tukey’s test.

Apoptosis and necrosis were examined by flow cytometry on an FACSCanto II cytometer. Figure 4 shows the percentages of apoptotic (panel B and E) and necrotic (panel C and F) breast cancer ZR-75-1 (panel B and C) and MDA-MB-231 (panel E and F) cells incubated for 48 h with rGO (100 µg/mL) and MG-132 (5 µM) or rGO (100 µg/mL) in combination with MG-132 (5 µM). We observed an increase in the number of apoptotic and necrotic cells incubated with rGO and MG-132 compared to untreated control cells. Interestingly, both breast cancer cell lines incubated with a mix consisting of rGO and MG-132 showed an increased percentage of apoptotic and necrotic cells compared to the tested cells incubated only with rGO or only with MG-132.

### 2.5. Cell Morphological Analysis

In order to validate the apoptosis and necrosis results obtained from the flow cytometer, analysis of cell morphological changes was performed under a fluorescence microscope, staining with ethidium bromide (EB) and acridine orange (AO). The fluorescence microscopy method distinguishes living cells (green nuclei, without chromatin condensation), apoptotic cells (green or red nuclei, with chromatin condensation) and necrotic cells (red nuclei, without chromatin condensation). 

**Figure 5:**
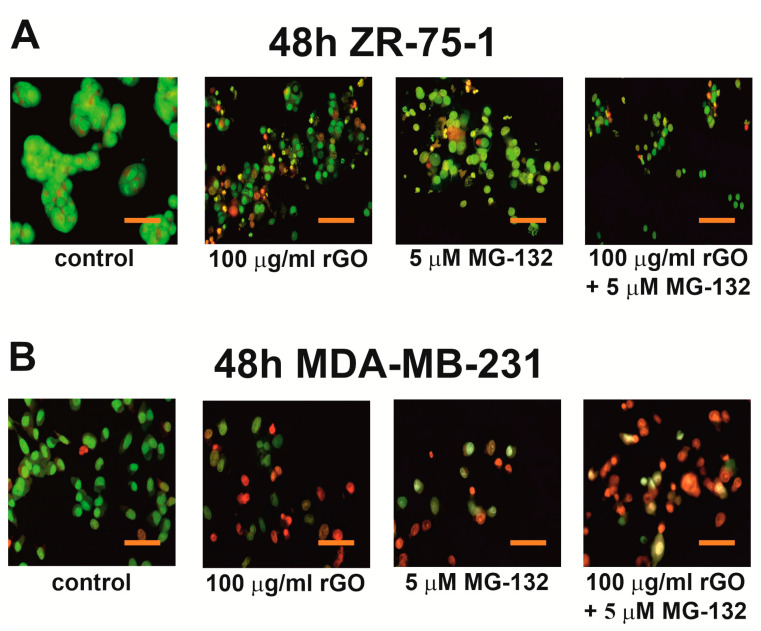
Effects of rGO, MG-132 or a mix of rGO and MG-132 on the apoptosis and necrosis of ZR-75-1 (panel **A**) and MDA-MB-231 (panel **B**) cells assessed by fluorescence microscope. Cells were incubated in a culture medium with rGO (100 µg/mL), MG-132 (5 µM) or a mix consisting of rGO (100 µg/mL) and MG-132 (5 µM) for 48 h and stained with EB/AO. Both breast cancer cells were imaged using a fluorescence microscope at 200-fold magnification and analyzed to identify viable and apoptotic cells. We present representative images from one of three independent experiments. Scale bar: 50 µm.

Figure 5 illustrates the morphological changes of apoptotic and necrotic cells. Figure 5A,B shows that rGO (100 µg/mL), MG-132 (5 µM) or a mix consisting of rGO (100 µg/mL) and MG-132 (5 µM) caused apoptosis induction in ZR-75-1 (Figure 5A) and MDA-MB-231 (Figure 5B) cells compared to untreated control cells. However, in the case of the ZR-75-1 and MDA-MB-231 cells incubated with a mix consisting of rGO and MG-132, the apoptosis and necrosis were more pronounced compared to cells incubated with only rGO or only MG-132. Apoptotic cells were characterized by condensation and marginalization of green or red nuclear chromatin. Necrotic cells were characterized by a red color without obvious condensation of nuclear chromatin. In particular, necrosis was observed in MDA-MB-231 cells rather than ZR-75-1 cells.

### 2.6. The Effect of rGO, MG-132 or Mix of rGO and MG-132 on Apoptosis Markers

**Figure 6:**
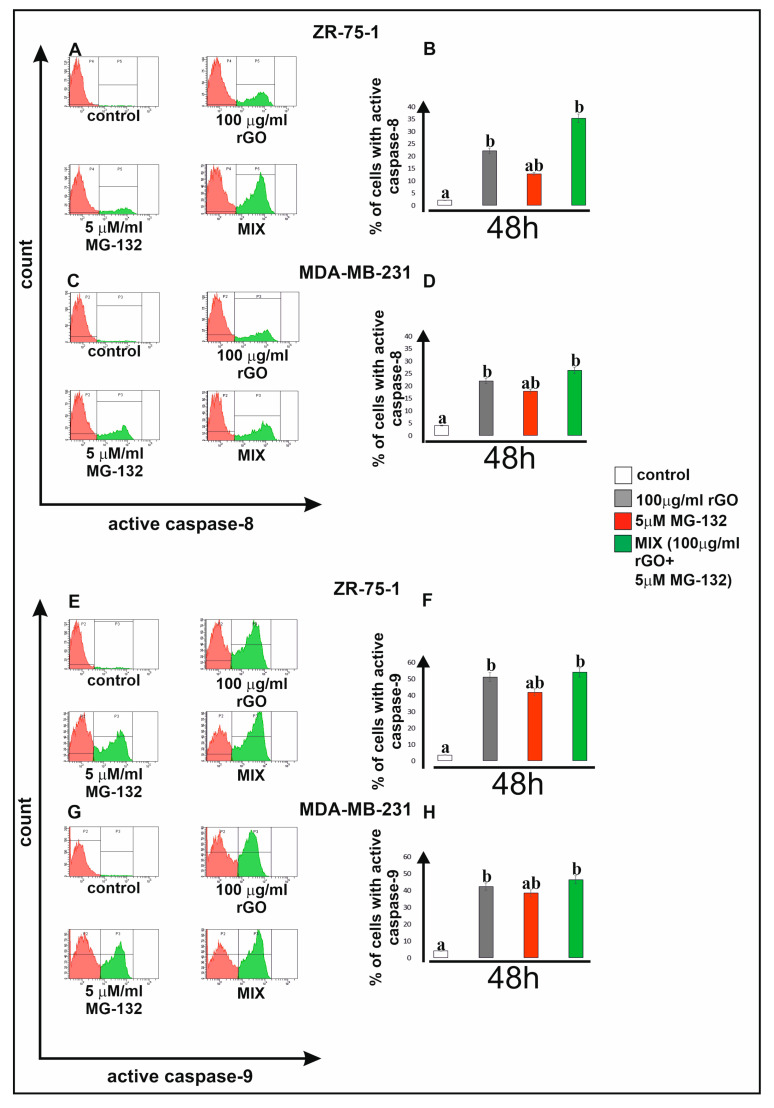
Flow cytometry analysis of active caspase-8 (**A**–**D**) and caspase-9 (**E**–**H**) in ZR-75-1 (**A**,**B**,**E**,**F**) and MDA-MB-231 (**C**,**D**,**G**,**H**) cell lines. Cells were treated with rGO (100 μg/mL), MG-132 (5 µM) or a mix consisting of rGO (100 μg/mL) and MG-132 (5 µM) for 48 h. Panels (**A**,**C**,**E**,**G**) show representative histograms of ZR-75-1 (**A**,**E**) and MDA-MB-231 (**C**,**G**) cells stained with FAM-FLICA caspase-8 or FAM-FLICA caspase-9. Panels (**B**,**D**,**F**,**H**) show the percentage of ZR-75-1 (**B**,**F**) and MDA-MB-231 (**D**,**H**) cells with active caspase-8 or active caspase-9. Gate P2—cell populations without active caspase-8 or caspase-9; gate P3—population of cells with active caspase-8 or caspase-9. Different letters indicate statistical differences (*p* ≤ 0.05) between treatments estimated by Tukey’s test.

Caspases play a key role in apoptosis. Caspase-8 mediates the extrinsic, while caspase-9 initiates the intrinsic apoptosis pathway. The effects of rGO (100 µg/mL), MG-132 (5 µM) or a mix consisting of rGO (100 µg/mL) and MG-132 (5 µM) on caspase-8 activity (Figure 6A–D) and caspase-9 activity (Figure 6E–H) were examined after 48 h. We observed an increase in active caspase-8 and active caspase-9 in cells incubated with rGO and MG-132 compared to the untreated control ZR-75-1 (Figure 6A,B,E,F) and MDA-MB-231 (Figure 6C,D,G,H) cells. Interestingly, both breast cancer cell lines incubated with a mix consisting of rGO and MG-132 showed increased percentages of active caspase-8 and active caspase-9 compared to the examined cells incubated with only rGO or only with MG-132.

### 2.7. The Effect of rGO on Morphological Changes of ZR-75-1 and MDA-MB-231 Cells

**Figure 7:**
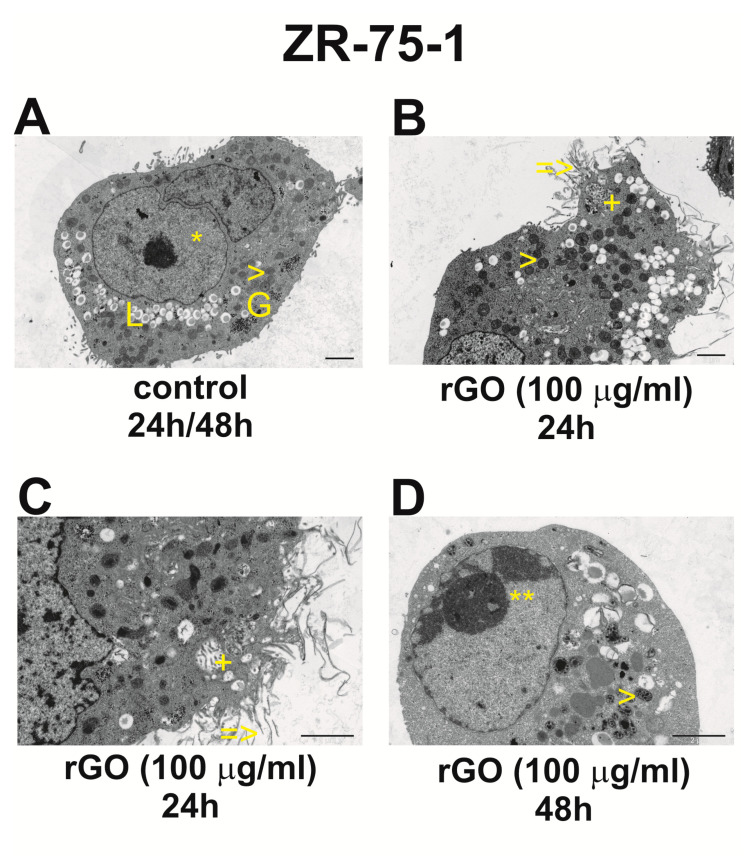
Interaction between rGO with the ZR-75-1 cells. Morphological changes in ZR-75-1 cells incubated with 100 μg/mL rGO for 24 h (**A**–**C**) and 48 h (**A**,**D**). Description of the symbols in the photos: On the cell surface there are few, medium-sized projections, and a cell nucleus with scattered chromatin (*) (**A**). Mitochondria with a variable electron matrix (>) (**A**,**B**,**D**), small lipid clusters (L) and glycogen (G) are also visible (**A**). A cell has rGO fibrils, which bulge out to form cavities (=>), present near the surface and tiny vesicles around the fibrils (**B**,**C**). In the cytoplasm, typical for this group of cells, cytoplasmic invaginations are visible in the form of the so-called ‘pseudocyst’ (+) (**B**,**C**), mitochondria with a dense matrix and an almost tubular arrangement of cristae (>) (**A**,**B**,**D**) and the nucleus with slightly clumped chromatin (**) (**D**). (**A**,**B**) – magnification 3000x, (**C**) – magnification 7000×, (**D**) – magnification 4400×.

**Figure 8:**
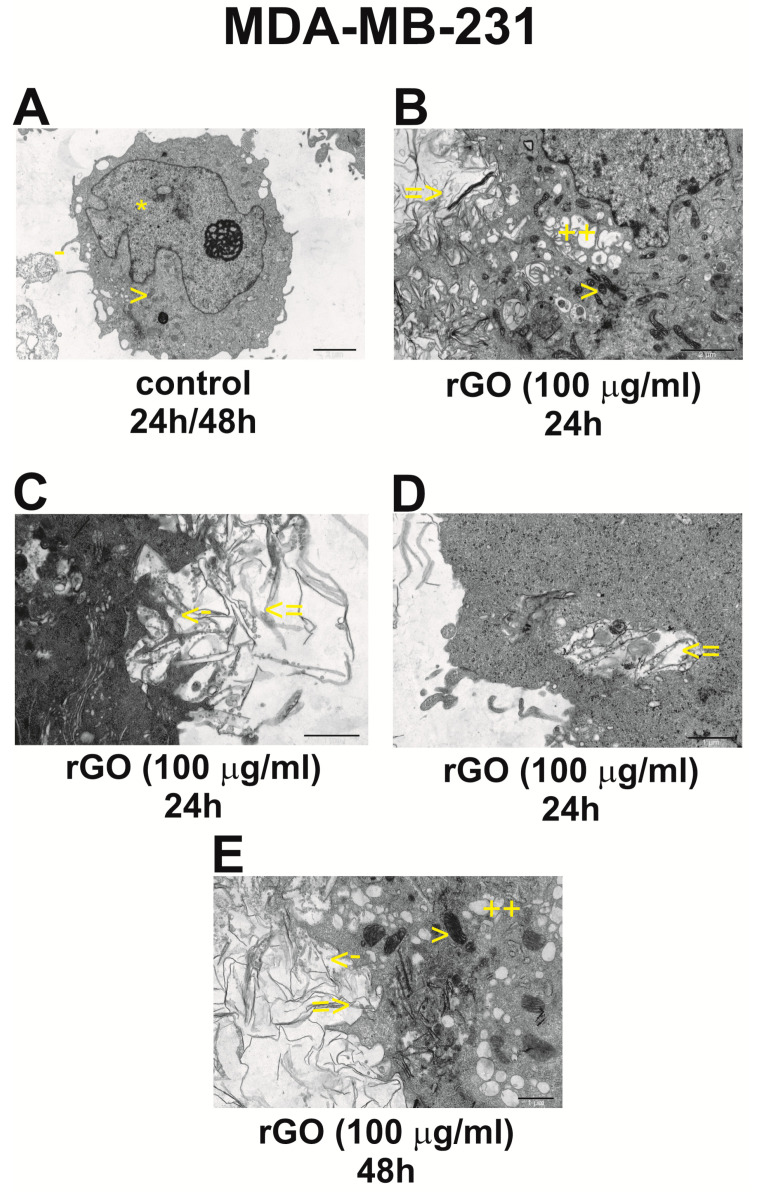
Interaction of rGO with MDA-MB-231 cells. Morphological changes in MDA-MB-231 cell lines incubated with 100 μg/mL rGO for 24 h (**A**–**D**) and 48 h (**A**,**E**). Cell surface with depressions and protrusions (-) (**A**), irregularly shaped cell nucleus with scattered chromatin and a large nucleolus (*) (**A**). Mitochondria with a medium-electron-density matrix (>) and few RER channels are also visible (**A**,**B**,**E**). There is a fragment of a cell with a highly irregular surface with numerous concavities in which graphene fibers are arranged (=>) (**B**–**E**). High electron density filaments are also visible within the cytoplasm (not surrounded by a membrane) (=>) (**B**–**E**). There are also mitochondria with a highly thickened matrix (>) (**A**,**B**,**E**), and strongly dilated cisternae of the Golgi apparatus (++) are also present (**B**,**E**). Numerous small vacuoles/vesicles are arranged on the surface of the fibrils (->) (**C**,**E**). (**A**) – magnification 3000×, (**B**) – magnification 4400×, (**C**–**E**) – magnification 7000×.

Figure 7 and Figure 8 show the morphological changes in the ultrastructure of breast cancer cell lines ZR-75-1 (Figure 7) and MDA-MB-231 (Figure 8) incubated with rGO (100 μg/mL) for 24 h and 48 h.

Figure 7A shows the morphology of control ZR-75-1 cells incubated for 24 h/48 h. These cells were characterized by a regular, round or oval shape, with a small number of small, finger-like projections resembling microvilli. Cell nuclei had various shapes, with highly dispersed chromatin and a condensed, distinct nucleolus. Small bundles of fibers were visible in the cytoplasm, around the cell nuclei. There were also oval mitochondria with a matrix of medium electron density, the Golgi apparatus, not very large glycogen clusters, short RER channels, numerous scattered ribosomes, and in many cells, small lipid vacuoles. ‘Pseudocysts’ were visible in the cytoplasm of many cells. There were round transparent spaces in which the cell membrane was covered with protrusions from the inside. Cells from the 48 h control group did not differ significantly from cells from the 24 h control group. Some cells had slightly larger glycogen clusters, and arc-shaped spaces appeared in the cytoplasm, surrounded by a single membrane.

These cells did not show any significant changes, and only those cells that were in contact with the fibrils had changed their cell membranes. Their typical small protrusions usually disappeared, and the surface often became smooth. The cell nuclei had more heterochromatin, and mitochondria had a different appearance. In some cells, they had a thickened mitochondrial matrix, but in many cells, they were clear with few crests. Many cells also had quite large accumulations of glycogen, while the lipid-containing vacuoles were often empty or partially used. In this group, there were few cells undergoing apoptosis and cells undergoing degeneration (Figure 7A).

Figure 7B,C show the morphological changes in the ultrastructure of the ZR-75-1 breast cancer cell line incubated with 100 μg/mL rGO for 24 h. 

In these cells, we observed a changed cell membrane surface, but this occurred only in the places where the fibrils adhered, while the remaining membrane, without contact with the fibrils, remained unchanged. Fragments of the cell membrane surrounded by fibrils had irregular cavities in which the filaments were arranged. Small bubbles were often visible on the surface of the fibrils located in the immediate vicinity of the cell membrane. In many cells, the following were observed: clumping of nuclear heterochromatin, differentiation of the size and shape of mitochondria, sometimes blurring of the crista membranes, sometimes thickening of the mitochondrial matrix, stimulation of the Golgi apparatus and the appearance of lysosomes nearby. A few apoptotically changed cells and cells with signs of degeneration, which were usually surrounded by fibrils, were also observed (Figure 7B,C).

Figure 7D shows the morphological changes in the ultrastructure of the ZR-75-1 breast cancer cell line incubated with 100 μg/mL rGO for 48 h. 

These cells did not show any significant changes, and only those cells that were in contact with the fibrils had changed cell membranes. Their typical small protrusions usually disappeared, and the surface often became smooth. The cell nuclei had more heterochromatin, and mitochondria had different appearance. In some cells, they had a thickened mitochondrial matrix, but in many cells, they were clear with a small number of crests. Many cells also had quite large accumulations of glycogen, while lipid-containing vacuoles were often empty or partially used. In this group, there were few cells undergoing apoptosis and cells undergoing degeneration (Figure 7D).

Figure 8A shows the morphology of control MDA-MB-231 cells incubated for 24 h/48 h.

We observed cells with a shape similar to round, with numerous, irregular protrusions on the surface. The cell nucleus was usually located eccentrically, with an irregular location, with a boundary, thickened with heterochromatin, scattered euchromatin and a localized nucleolus. Oval or elongated mitochondria containing an electron-regular matrix were present in the cytoplasm, and occasional bright spots were visible in some organelles. Also visible were short and inaccessible rough endoplasmic networks, scattered ribosomes and sparse glycogen granules, Golgi devices and fibers. Arc-shaped spaces were sometimes visible in the thin cytoplasm, surrounded almost along the entire length by a single membrane. Cells were sometimes found in the process of dividing a set. After 48 h, we observed cells with a slightly more irregular surface compared to the 24 h control group, with long, differently shaped projections. Cell nuclei had very irregular contours, with large cytoplasmic invaginations, scattered euchromatin and prominent nucleoli. The remaining cellular structures were similar to those in the control group after 24 h (Figure 8A). 

Figure 8B–D show the morphological changes in the ultrastructure of the MDA-MB-231 breast cancer cell line incubated with 100 μg/mL rGO for 24 h.

In this group, numerous cells surrounded by rGO fibrils were observed. In the places where the fibrils adhered, the surface of these cells was usually highly irregular with numerous indentations and protrusions, and the fibrils were arranged in the depressions. Sometimes, in such places, segmental blurring of the structure or damage to the cell membrane was visible. Sometimes, numerous small vacuoles, similar in size to pinocytic vesicles, were visible on the surface of the rGO fibrils. In some cells, filaments were visible within the cytoplasm. Usually, such a space was slightly isolated from the remaining cytoplasm, with a single membrane appearing in sections. In mitochondria, a highly thickened mitochondrial matrix and clear intermembrane spaces were visible. Cell nuclei showed moderate clumping of heterochromatin. The Golgi apparatus usually had greatly dilated cisternae. The remaining organelles did not show any significant deviations from the norm (Figure 8B–D).

Figure 8E shows the morphological changes in the ultrastructure of the MDA-MB-231 breast cancer cell line incubated with 100 μg/mL rGO for 48 h. In this group, cells with a very irregular surface, with numerous protrusions and cavities in which graphene fibers were arranged, were observed. Small bubbles were observed around the fibrils or on their surface. Sometimes sections of damaged cell membrane with fibrils were visible, and sometimes filaments were found within the cytoplasm. Often, the area of the cytoplasm containing the fibrils was isolated from the rest of the cell’s cytoplasm by a single membrane. In most cells, mitochondria with a highly condensed mitochondrial matrix and Golgi apparatus, in the form of numerous enlarged cisternae, and lysosomes were visible. Shortening of RER channels and, in places, shedding of ribosomes (RER degranulation) were visible. Apoptotically changed cells and degenerating cells were also found. In these cases, very numerous fibrils were present mixed with damaged, highly whitened cell organelles (Figure 8E).

## 3. Discussion

Cancer diagnosis and treatment are currently one of the leading goals of biomedical research. Standard therapeutic techniques used in the fight against breast cancer do not bring the expected results. There may be many reasons for therapeutic failure. One of them is the promotion of the development of cancer cells by activating pathways that promote tumor division and proliferation. Due to the uncontrolled growth of cancer cells and unnatural gene expression, cancers have many features that distinguish them from normal cells. These include the low pH of the cytoplasm of cancer cells, overexpression of metalloproteinases (MMPs) and high levels of ROS. Moreover, the cytoplasm of cancer cells is rich in ATP and GSH. Therefore, innovative therapeutic strategies are being sought in the fight against breast cancer. One of the possible therapeutic strategies is the use of nanotechnology in the fight against breast cancer. The use of reduced graphene oxide and a proteasome inhibitor (MG-132) opens new possibilities in breast cancer therapy. 

In our studies, we showed that rGO and MG-132 induce cytotoxic effects on breast cancer cells. Moreover, the combination of rGO with MG-132 enhances this effect. Hydrophobic forms of graphene have a high ability to bind to alpha-helical areas in the structure of peptides, which is due to the curvature of the surface of its molecule, which favors such interactions. In contrast, the proteasome inhibitor MG-132 can effectively inhibit the proteolytic activity of the 26S proteasome complex and induces strong oxidative stress leading to cytotoxicity. It has been shown that the sharp edges of rGO can mechanically damage cell membranes, leading to the release of LDH from cells [10,11]. The ROS cause lipid pre-oxidation, and thus, large amounts of LDH are released into the culture medium. Li et al. showed the release of LDH into a culture medium could cause rGO-induced loss of cell membrane integrity [12]. The combination of a proteasome inhibitor MG-132 and rGO leads to increased cytotoxic effects on breast cancer cells. Ma et al. indicated that graphene oxide inhibits the activity of the 20S proteasome in living cells. Researchers indicate that GO can inhibit the proteasomal degradation of proteins involved in cell cycle regulation, such as P21. The accumulation of P21 contributes to cell cycle arrest in G_0_/G_1_. The cytotoxic effect of GO on cells is related to the inhibition of the proteasome activity of the α-subunit. In this situation, when GO blocks the entry and exit of the protein substrate, the action of the proteasome catalytic subunits is blocked. Therefore, both rGO and MG-132 may have a similar mechanism of cytotoxic effect on breast cancer cells by inhibiting proteasomal activity [13]. Apart from that, at concentrations above 75 µg/mL, pure graphene adheres to the surface of RAW264.7 cells, which causes unnatural stretching of cell membranes. This can cause the cell membrane to rupture and release LDH. rGO can interact with cell membrane receptors and disturb its metabolism by inhibiting the transport of glucose and amino acids into the cells. This leads to endoplasmic reticulum stress (ER stress), apoptosis or autophagy. Due to its spatial structure, rGO can capture glucose and amino acids like a molecular sieve, thus limiting the access of nutrients to cells [11]. Moreover, the combination of a proteasome inhibitor (MG-132) with rGO may enhance the effect of ER stress. Krętowski et al. showed that blocking the action of proteasomes, by bortezomib, leads to a reduction in the expression of the chaperone GRP170 and nuclear factor NF-kB. All these processes may lead to decreased cell proliferation and increased cytotoxicity [14].

MG-132 acted as a stress factor, significantly reducing breast cancer viability. Lee et al. indicated that MG132 suppressed U2OS cell proliferation. Furthermore, it was found that MG132 treatment increased apoptosis and induced DNA damage in U2OS cell lines [15].

Studies indicate that small GOs enter cells mainly through clathrin-dependent endocytosis, while increasing the size of GO intensifies their uptake via phagocytosis [11]. Chatterjee and colleagues showed that GO could be internalized by HepG2 cells; however, reduced graphene oxide (rGO), which is much more hydrophobic compared to GO, adsorbs on the cell surface without internalization [7]. Furthermore, in some cell types, both GO and rGO of different sizes can also be taken up by cells via endocytosis [11], whereas in our study, we indicated that MDA-MB-231 cells incubated in the presence of rGO are damaged in contact with rGO fibrils, and cells around which there are no rGO fibrils do not show any significant deviations compared to the control group. rGO fibrils were visible in the cytoplasm, but only in the MDA-MB-231 cells, as they were not visible in ZR-75-1 cells. Often, rGO fibrils were isolated from the rest of the cytoplasm by a single membrane. In the breast cancer ZR-75-1 cells, rGO did not cause such damage to the cell membrane as in MDA-MB-231 cell lines. This may indicate greater sensitivity of MDA-MB-231 cells to rGO. An electron microscope image shows that the disappearance of small protrusions and the smoothing of the cell membrane did not allow rGO fibrils to penetrate the cell.

According to the literature data, rGO and MG-132 may change human cellular metabolism by influencing oxidative stress processes. The main factors that induce oxidative stress in breast cancer cells incubated in the presence of MG-132 and rGO are reactive oxygen species (ROS) [10,11]. ROS play a pivotal role in various biological processes. ROS include hydrogen peroxide (H_2_O_2_), superoxide anion (O_2_•^−^) and hydroxyl radical (•OH), which play an important role in cellular metabolism, signaling, activation of transcription factors, modulation of gene expression, differentiation, cell proliferation and homeostasis [11]. It has been proven that reactive oxygen species are important factors involved in both the induction of cancer transformation and the development and progression of cancer. The increased content of ROS in cancer cells compared to healthy cells may be caused by excessive proliferation, DNA mutations or other dysfunctions. Up to a certain level, oxidative stress stimulates the growth and proliferation of cancer cells, but once this level is exceeded, it becomes lethal to them. Therefore, it is very desirable to induce oxidative stress in cancer cells, which will direct them towards apoptosis without damaging healthy cells. In our research, in addition to reduced graphene oxide, we also used the compound MG-132, which, as it turned out, acted synergistically with reduced graphene oxide, clearly increasing the level of ROS in the cells of both tested cell lines. Our results obtained as a result of research on both breast cancer cell lines are consistent with literature data clearly indicating the stimulating effect of graphene on the increase in the level of oxidative stress. It should be noted that in our experiment, the effect of reduced graphene oxide was somehow enhanced by the addition of MG-132. Both Gurunathan et al. and Zhou et al. showed cytotoxic effects of graphene oxide on the estrogen-dependent MCF-7 cell line and the estrogen-independent MDA-MB-231 cell line [15,16]. However, according to Zhang et al. and Jarosz et al., rGO showed even higher toxicity than GO. This may be due to the fact that GO, compared to rGO, is characterized by hydrophilic properties, smoother edges and higher oxygen content. All these factors may reduce the toxicity of GO in relation to rGO. Research results presented in the literature indicate a high affinity of rGO for cell membranes, which may subsequently induce apoptosis [17,18]. Overproduction of ROS, also observed in our experiment, may adversely affect the stability of nucleic acids, reduce the activity of antioxidant enzymes and, as we also observed, increase the MDA content and decrease the level of SH groups. This leads to an increase in the level of lipid peroxidation, a decrease in the content of thiols and, consequently, oxidative damage to cells [19]. Excessively high levels of ROS are associated with changes in the mitochondrial membrane potential, which in turn causes mitochondrial dysfunction and, consequently, the induction of apoptosis [20,21]. In our studies, we observed a similar situation as a result of exposure of both tested lines to rGO and to both tested compounds (rGO and MG-132) simultaneously.

Enzymatic antioxidants such as SOD and GPx belong to the endogenous antioxidant defense system [21]. The presented results clearly show the induction of an increase in oxidative toxicity in breast cancer cells treated with both rGO and MG-132 and a mixture of these compounds. The excessive ROS synthesis induced by rGO or MG-132 causes a significant decrease in GSH synthesis and stimulates the activity of antioxidant enzymes such as SOD [21].

Literature data report that increased levels of GSH in cells promote cancer initiation and progression [22]. Therefore, the aim is mainly to decrease the level of reduced glutathione and the level of thiol group content in order to inhibit the proliferation of breast cancer cells and therefore inhibit the development of cancer. In our study, we demonstrated the effect of rGO and MG132 on reducing the level of thiol groups and reduced glutathione.

Chatterjee et al. showed that GO/rGO induces apoptosis in HepG2 cells. Moreover, these researchers showed that the tested nanostructures induce an increase in the level of Bax protein and a decrease in the level of Bcl-2 protein. The induction of apoptosis was observed simultaneously with the intensification of oxidative stress in HepG2 cells induced through GO/rGO [7]. Similar results were presented by Zheng et al. Researchers have shown that MG-132 induces ROS synthesis and at the same time leads to an increase in the level of Bax and p53 proteins and a decrease in the level of Bcl-2 protein in OSCC cells [10]. In our previous study, we indicated that rGO could induce apoptosis through the oxidative stress pathway in ZR-75-1 and MDA-MB-231 cell lines. We observed that rGO induced an increase in Bax protein levels and a decrease in Bcl-2 and Bcl-xL protein levels. Apart from that, Krętowski et al. observed that rGO arrests the cell cycle and increases the level of p21 protein in breast cancer cells [9]. Increases in ROS production may lead to mitochondrial damage and disruption of the mitochondrial membrane potential (ΔΨ_m_) of cancer cells [11]. In our study, we conducted an ultrastructural analysis of MDA-MB-231 and ZR-75-1 cells. We have shown that mitochondria often have a very dark (electron-dense) matrix, but there is great variation in the structure of mitochondria depending on the degree of activity and contact/damage by rGO fibrils. This may indicate reduced mitochondrial membrane potential. In our previous study, we indicated that rGO can decrease mitochondrial membrane potential (MMP) and changes in apoptotic cell morphology [9]. Li et al. were the first to present that graphene can induce a decrease in the MMP and increase the production of ROS, which activate the mitochondria-dependent apoptotic pathway [12]. When cells are incubated with graphene, the outer membrane of mitochondria is damaged, cytochrome c is released and caspase activity is increased, ultimately leading to cell death [11]. Additionally, we observed an increase in active caspase-8 and active caspase-9 in the cells incubated with rGO and MG-132 compared to the untreated control ZR-75-1 and MDA-MB-231 cells. Interestingly, both breast cancer cell lines incubated with a mix consisting of rGO and MG-132 showed increased percentages of active caspase-8 and active caspase-9 compared to examined cells incubated with rGO or with MG-132. The most important caspases involved in apoptosis are caspase-8, caspase-9 and caspase-3. Caspase-8 mediates the extrinsic effect, while caspase-9 initiates the intrinsic apoptosis pathway [23]. Chatterjee et al. indicated that rGO could induce the caspase-8 expression gene without changing the caspase-9 and caspase-3 expression genes, while GO increases the expression of caspase-9 and caspase-3 but not caspase-8 [7]. Furthermore, Lee et al. showed that MG-132 can decrease the expression level of anti-apoptotic proteins CDK2, CDK4, Bcl-xL and Bcl-2, while increasing the expression level of pro-apoptotic proteins p21; p27; p53; P-p53; cleaved forms of caspase-3, caspase-7 and caspase-9; PARP; and FOXO3 in U2OS cell lines [5]. Our research shows that rGO and MG-132 can simultaneously induce receptor and mitochondrial apoptosis pathways.

The multitude of data presented in Table 1 confirms the need to conduct and develop research in the direction presented in this work. These data constitute the basis for subsequent, planned experiments, also conducted in vivo, aimed at developing new, effective therapeutic methods.

## 4. Materials and Methods

### 4.1. Reagents

Media and reagents for culture cells: L-15 Medium (1×) + GlutaMAX^TM^-I, trypsin-EDTA, RPMI Medium 1640 (1×) + GlutaMAX^TM^-I, DPBS, penicillin, streptomycin and fetal bovine serum Gold (FBS Gold) were provided by Gibco (San Diego, CA, USA). Kits for apoptosis detection: FITC Annexin V apoptosis detection Kit I was obtained from BD Pharmingen^TM^ (San Diego, CA, USA); FAM-FLICA Caspase-8 and FAM-FLICA Caspase-9 Assay were provided by ImmunoChemistry Technologies (Davis, CA, USA). Chemical treatment of cells: reduced graphene oxide (rGO) was obtained from Sigma-Aldrich (St. Louis, MO, USA), while the proteasome inhibitor (MG-132) was provided by Selleckchem (Cologne, Germany). Kit for cytotoxicity analysis: the LDH-Cytotoxicity Assay kit was provided by BioVision (St. Louis, CA, USA). The compounds for oxidative stress analysis: SDS, TCA, TBA, Folin–Ciocalteu reagent and 2′,7′-dichlorodihydrofluorescein diacetate (H_2_DCFDA) were provided by Sigma-Aldrich (St. Louis, MO, USA), and GSH/GSSG-Glo^TM^ Assay kit by Promega (Madison, WI, USA). The compounds for ultrastructure analysis: glutaraldehyde, paraformaldehyde, cacodylate buffer, osmium tetroxide, ethanol and glycid ether were provided by Sigma-Aldrich (St. Louis, MO, USA).

### 4.2. Cell Culture

Human breast cancer cell lines MDA-MB-231 and ZR-75-1 were obtained from the American Type Culture Collection (ATCC). MDA-MB-231 cells were cultured in L-15 Medium (1×) + GlutaMAX^TM^-I, and ZR-75-1 cells were cultured in RPMI Medium 1640 (1×) + GlutaMAX^TM^-I, while the human skin fibroblasts were cultured in DMEM (1×) + GlutaMAX^TM^-I. The media were supplemented with 10% heat-inactivated fetal bovine serum GOLD (FBS GOLD), penicillin (100 U/mL) and streptomycin (100 µg/mL). Cells were cultured in Falcon flasks (BD Pharmingen^TM^, San Diego, CA, USA) in a Galaxy S+ CO_2_ incubator (RS Biotech, Irvine, UK) at 37 °C and 5% CO_2_ (or without CO_2_-MDA-MB-231) and 95% air in a Galaxy S+ incubator (RS Biotech, Irvine, UK). At approximately 70% confluence, cells were detached with 0.05% trypsin and 0.02% EDTA and counted in a Scepter Cell Counter (Millipore, MA, USA). Then, 2.5 × 10^5^ cells per well were seeded in 2 mL of L-15 Medium (1×) + GlutaMAX^TM^-I or RPMI Medium 1640 (1×) + GlutaMAX^TM^-I in six-well plates for 24 h. 

### 4.3. Chemical Treatment of Breast Cancer Cells

Graphene oxide (GO) is a monolayered carbon structure with oxygen-containing functional groups (epoxide, carbonyl, carboxyl and hydroxyl) attached to both sides of the layer as well as the edges of the plane. Many modern procedures for the synthesis of GO are based on the method first reported by Hummers in which graphite is oxidized by a solution of potassium permanganate in sulfuric acid. When the electrically insulating GO is reduced, the reduced graphene oxide (rGO) formed resembles graphene but contains residual oxygen and other heteroatoms as well as structural defects [32,33,34]. The X-ray analysis showed diffraction conforming to the structure of rGO. The carbon content was 81%, and oxygen 17%.

To avoid the problem of aggregation, reduced graphene oxide (rGO) was dispersed in deionized water (1 mg/mL) by a sonicator, Sonopuls (Bandelin, Berlin, Germany), on ice for 45 min (160 W, 20 kHz). During experiments, the cell culture media were replaced with a new medium containing rGO suspensions, at concentrations ranging from 25 μg/mL to 200 μg/mL, or MG-132 (5 µM) or a mix consisting of rGO (100 μg/mL) plus MG-132 (5 µM). The negative control constituted untreated cells. For the analysis of cell viability, the rGO concentration range was from 25 to 200 µg/mL. Subsequently, cells were exposed to rGO for 3 h, 6 h, 12 h, 24 h and 48 h. Apoptosis, necrosis and biochemical parameters of oxidative stress detection were assessed under the effect of rGO (100 μg/mL), MG-132 (5 µM) or a mix consisting of rGO (100 μg/mL) and MG-132 (5 µM). For the analysis of caspase-8 and caspase-9 enzymatic activity, cells were treated with rGO (100 μg/mL), MG-132 (5 µM) or a mix consisting of rGO (100 μg/mL) and MG-132 (5 µM) for 48 h. Apart from that, for the ultrastructure analysis, both breast cancer cell lines were treated with rGO (100 μg/mL) for 24 h and 48 h.

### 4.4. Cell Viability

A lactic dehydrogenase (LDH) test was used to evaluate cell membrane integrity. The breast cancer cell lines were plated in 96-well plates (1 × 10^4^ cells per well) in 200 μL of medium and incubated for 24 h. After 24 h, the medium was removed and replaced with the rGO suspension in a medium at 25 to 200 μg/mL concentrations, which was incubated for 3 h, 6 h, 12 h, 24 h and 48 h. A total of 100 μL of the lactate dehydrogenase assay mixture was added to each well. The culture was covered and incubated for 20 min at RT. The OD was recorded as outlined, and the LDH leakage was expressed as a percentage of OD. If the cell membrane is damaged, intracellular LDH molecules are released into the culture medium. The LDH level in the medium indicates cell membrane damage.

### 4.5. Detection of Apoptosis and Necrosis

Apoptosis and necrosis of breast cancer cell lines were evaluated by flow cytometry on an FACSCanto II cytometer (BD, San Diego, CA, USA). MDA-MB-231 and ZR-75-1 cells (2.0 × 10^5^ per well) were seeded in 2 mL of medium in six-well plates. After 24 h, the medium was removed and replaced with the rGO suspension in a medium at 100 μg/mL, MG-132 (5 µM) or a mix consisting of rGO (100 μg/mL) and MG-132 (5 µM). All cell lines were incubated for 48 h. The cells were detached, resuspended in a medium and then resuspended in binding buffer. Subsequently, the cells were stained with FITC Annexin V and PI (FITC Annexin V apoptosis detection Kit I, (BD Pharmingen^TM^, San Diego, CA, USA) at room temperature, in the dark, for 15 min. Data were analyzed using FACSDiva software, Ver. 6.1.3 (BD Pharmingen^TM^, San Diego, CA, USA), and 10.000 cells were measured per sample.

### 4.6. The Cell Morphological Analysis

Cells were stained with fluorescent dyes, including acridine orange and ethidium bromide, in order to evaluate the nuclear morphology of apoptotic cells. MDA-MB-231 and ZR-75-1 cells grew on a cover glass with rGO (100 µg/mL), MG-132 (5 µM) or a mix consisting of rGO (100 μg/mL) and MG-132 (5 µM) for 48 h. Thereafter, the cells were washed twice with 1× DPBS and stained with 1 mL of the dye mixture (10 μM acridine orange and 10 μM ethidium bromide in 1× DPBS) for 10 min in the dark at room temperature (RT). Next, the stained solution was removed, and the cells were washed with 1× DPBS, analyzed and imaged under a fluorescence microscope at 200× magnification. Acridine orange is a vital dye that will stain both live and dead cells, whereas ethidium bromide will stain only cells that have lost their membrane integrity. Each sample was analyzed by fluorescence microscopy (Olympus CXK41, U-RLFT50, Tokyo, Japan) according to the following criteria: living cells—normal green nucleus; early apoptotic cells—bright green nucleus with condensed or fragmented chromatin; late apoptotic cells—orange-stained nuclei with chromatin condensation or fragmentation. 

### 4.7. Caspase-8 and Caspase-9 Enzymatic Activity Assay

Caspase-8 and caspase-9 activity was measured using the FAM-FLICA Caspase-8 or Caspase-9 Kit (ImmunoChemistry Technologies, Davis, CA, USA) according to the manufacturer’s instructions. MDA-MB-231 and ZR-75-1 cells were treated with 100 µg/mL rGO, MG-132 (5 µM) or a mix consisting of rGO (100 μg/mL) and MG-132 (5 µM). or 48 h. Next, the cells were harvested and washed with cold 1× DPBS. Then, 10 µL of diluted FLICA reagent was added to 290 µL of the cell suspension. The cells were incubated at 37 °C for 60 min. After incubation, the cells were washed twice with 400 µL of buffer and centrifuged at 1000× *g* for 5 min. Analysis was performed using a BD FACSCanto II (San Diego, CA, USA) flow cytometer, and results were analyzed with FACSDiva software.

### 4.8. Intracellular ROS Detection

The level of intracellular ROS was determined using the dichlorodihydrofluorescein diacetate (DCFH-DA) assay (Sigma, St. Louis, MO, USA). After diffusion through the cell membrane, DCFH-DA is deacetylated by cellular esterases to a non-fluorescent compound, which is later oxidized by intracellular ROS into fluorescent 2′,7′-dichlorofluorescein (DCF). MDA-MB-231 and ZR-75-1 cells (1 × 10^4^ cells per well) were seeded in 200 μL of DMEM or RPMI in 96-well black plates. After 24 h, DMEM or RPMI was removed, and the cells were stained with 10 μM of DCFH-DA in PBS at 37°C, 5% CO_2_, for 45 min. Then, the dye was removed and replaced with the rGO suspensions in DMEM or RPMI at 100 μg/mL, MG-132 (5 µM) or a mix consisting of rGO (100 μg/mL) and MG-132 (5 µM) and incubated for 48 h. The DCF fluorescence intensity was measured by Infinite M200 microplate reader (Tecan, Männedorf, Switzerland) at the excitation wavelength of 485 nm and the emission wavelength of 535 nm. The intracellular ROS generation in breast cancer cells stimulated by rGO, MG-132 (5 µM) or a mix consisting of rGO (100 μg/mL) and MG-132 (5 µM) was shown as the intensity of fluorescence of the DCF.

### 4.9. Determination of SH Groups

The SH groups were measured using the method of Rice-Evans [35], as described previously by Jabłońska-Trypuć et al. [36]. MDA-MB-231 and ZR-75-1 cells (2.5 × 10^5^ cells/mL) were incubated in 2 mL of a medium with rGO (100 μg/mL), MG-132 (5 µM) or a mix consisting of rGO (100 μg/mL) and MG-132 (5 µM) or without compounds in 6-well tissue culture plates. All the experiments were performed in triplicate.

### 4.10. Determination of TBA Reactive Species (TBARS) Levels

The level of membrane lipid peroxidation products, or TBARS, was measured using the method of Rice-Evans [35], as described previously by Jabłońska-Trypuć et al. [36]. MDA-MB-231 and ZR-75-1 cells (2.5 × 10^5^ cells/mL) were incubated in 2 mL of medium with or without the test rGO (100 μg/mL), MG-132 (5 µM) or mix consisting of rGO (100 μg/mL) and MG-132 (5 µM) in 6-well tissue culture plates. All the experiments were performed in triplicate.

### 4.11. Determination of GSH/GSSG

Total glutathione and the GSH/GSSG ratio were each assayed in triplicate via the GSH/GSSG-Glo™ kit (Promega, Madison, WI, USA) following the manufacturer’s instructions. MDA-MB-231 and ZR-75-1 cells were seeded in white-bottom 96-well plates at 10^4^ cells/well (Sarstedt); allowed to attach; and treated with rGO (100 μg/mL), MG-132 (5 µM) or a mix consisting of rGO (100 μg/mL) and MG-132 (5 µM). Prior to the assay, growth media were removed, and cells washed with PBS. The assay is based on a luminescence measurement and detects and quantifies total glutathione (GSH + GSSG), GSSG and GSH/GSSG ratios in cultured cells. Stable luminescent signals are correlated with either the GSH or GSSG concentration of a sample. In this method, GSH-dependent conversion of a GSH probe, Luciferin-NT, to luciferin by a glutathione S-transferase enzyme is coupled to a firefly luciferase reaction. Light from luciferase depends on the amount of luciferin formed, which in turn depends on the amount of GSH present. Thus, the luminescent signal is proportional to the amount of GSH. GSH/GSSG ratios are calculated directly from luminescence measurements.

### 4.12. Determination Glutathione Peroxidase Activity

Glutathione peroxidase activity was studied according to Jabłońska-Trypuć et al. [37]. ZR-75-1 and MDA-MB-231 cells were incubated in six-well plates at the density of 1 × 10^5^ cells/well (Sarstedt, Germany, Numbrecht), and they were treated with different compounds for 48 h. For the determination of glutathione peroxidase activity, the GPx Assay kit (Cayman Chemical Company, Ann Arbor, MI, USA) was used following the manufacturer’s instructions. The absorbance was read at 340 nm using the GloMax^®^-Multi Microplate Multimode Reader (Promega, Madison, WI, USA). All the experiments were performed in triplicate. 

### 4.13. Determination Superoxide Dismutase Activity

Superoxide dismutase activity was studied according to Jabłońska-Trypuć et al. [37]. Cells were incubated in six-well plates at 1×10^5^ cells/well (Sarstedt), and they were treated with different compounds for 48 h. For the determination of SOD activity, the Superoxide Dismutase Assay Kit (Cayman Chemical Company, Ann Arbor, MI, USA) was applied following the manufacturer’s instructions. The absorbance (440–460 nm) was read using the GloMax^®^-Multi Microplate Multimode Reader. All the experiments were performed in triplicate.

### 4.14. Transmission Electron Microscopy

The morphological changes in human ZR-75-1 and MDA-MB-231 cells were evaluated by transmission electron microscopy (TEM). Both cell lines (2.5 × 10^5^) were seeded in 2 mL of a medium in six-well plates. After 24 h, the medium was removed and replaced by the rGO suspensions in a medium at the concentration of 100 μg/mL. Subsequently, the ZR-75-1 and MDA-MB-231 cells were incubated for 24 h and 48 h. After incubation, the cells were centrifuged (1000× *g*, 5 min), fixed in a mixture of 2.5% glutaraldehyde and 2% paraformaldehyde in 0.1 M cacodylate buffer (CB) at pH 7.0, at 4 °C, for 1 h, and taken up into the agar blocks. Then, samples were washed in CB at 4 °C, for 1 h, post-fixed in 1% osmium tetroxide in CB at 4 °C for 1 h and next dehydrated through a graded series of ethanol and embedded in glycid ether 100 (Epon 812). Ultrathin sections were contrasted with uranyl acetate and lead citrate, mounted on nickel grids and evaluated in an OPTON 900 transmission electron microscope (Zeiss, Oberkochen, Germany).

### 4.15. Determination of Total Protein Concentration

Protein concentration was determined spectrophotometrically according to the method of Lowry [38].

### 4.16. Statistical Analysis

Differences between treatments and untreated control human cells were analyzed by one-way ANOVA, followed by Dunnett’s procedure for multiple comparisons. Significant effects are represented by *p* ≤ 0.05 (*). Significant differences were estimated by the Tukey test at *p* < 0.05. We reported data as means ± SD from three independent measurements and analyzed the results using one-way ANOVA with Scheffe’s F test. A * *p* ≤ 0.05 was considered statistically significant.

## 5. Conclusions

In conclusion, we suggest that rGO, MG-132 and their combination can induce cytotoxicity in ZR-75-1 and MDA-MB-231 breast cancer cell lines. We observed these two cell lines exhibit increased oxidative stress and the upregulation of apoptosis, both the extrinsic and intrinsic pathways. Apart from that, we found that treatment with rGO, MG-132 or the mix increased apoptosis, necrosis and induction of caspase-8 and caspase-9 activity in ZR-75-1 and MDA-MB-231 cells. Apoptosis and caspase activation were accompanied by changes in the ultrastructure of mitochondria in both breast cancer cells incubated with rGO. These results may indicate that the combination of rGO and MG-132 is a promising therapeutic option in the fight against breast cancer cells.

## Figures and Tables

**Table 1:** The effect of rGO and MG-132 on oxidative stress and apoptosis in different cell lines.

Material	Nanomaterial Size	Cell Types	Exposure Conditions	Effect	Reference
rGO	0.5–3 µm	A549	200 µg/mL, 24 h	Significant decrease in cell viability.	[24]
rGO	0.4–0.8 µm	HUVEC	10 µg/mL	rGO induces significant increases in intracellular ROS production and mRNA levels of HO1 and TrxR.	[25]
rGO	-	A549	0.1225–12.5 µg/cm^2^ for 5 days	The cells incubated with lower concentrations of rGO did not lead to increases in ROS synthesis.	[26]
rGO	40 nm	HepG2	1–200 mg/L for 24 h	The rGO was found to be mostly adsorbed at the cell surface without internalization. ROS synthesis by physical interaction.	[7]
rGO	-	MSC	0.01–100 µg/mL for 24 h	Destruction of the cells with the threshold concentration of 1 mg/mL, while cytotoxicity of the rGO sheets appeared at high concentrations of 100 mg/mL after 1 h.	[27]
rGO	100 nm to 1.5 µm	U87 U118-MG	100 µg/mL	rGO enters glioblastoma cells and reduces cell viability and proliferation with an increase in dose. The percentage of apoptotic cells increased in rGO-treated glioma cells.	[28]
MG-132	-	MCF-7	-	MG-132 induced apoptosis and autophagy in MCF-7 cells. Apart from that, the proteasome inhibitor induced an increase in caspase-3 level and Bax and decrease in Bcl-2 level.	[29]
MG-132	-	OS-RC-2	0.01–1 µM	MG-132 decreased cell viability and induced apoptosis of cells. Apart from that, the proteasome inhibitor induced an increase in the level of ROS synthesis.	[30]
MG-132	-	Cancer cells	-	MG-132 induces apoptosis by the formation of ROS.	[2]
MG-132	-	Rat C6 glioma	-	MG-132 decreased cell viability and induced apoptosis of cells by ROS synthesis. Western blot analysis revealed that MG-132 induced downregulation of anti-apoptotic proteins Bcl-2 and XIAP, upregulated expression of pro-apoptotic protein Bax and caspase-3, and produced cleaved C-terminal 85 kDa PARP.	[4]
MG-132	-	A549	-	MG132 inhibited the growth of human A549 cells by inducing cell cycle arrest as well as triggering apoptosis, which was in part correlated with changes in ROS and GSH levels.	[31]

## Data Availability

The data presented in this study are available on request from the corresponding author.
